# Indents

**Published:** 1914-11-15

**Authors:** 


### INDENTS.

**☞Brief Articles of Technical or Scientific Value will receive notice and comment. Contributions invited.**

*Ten Years Progress in Medical Education.*

The following facts will show in a measure the progress made in medical education during the first decade of the existence of the Council on Medical Education.

(*a*) Instead of having 160, or one-half of the world's supply of medical schools, this country now has about 100, and these have been greatly improved in every respect.

(*b*) Instead of only four medical colleges requiring one or more years of college work for admission there are now eighty-two which either have already put that requirement into effect or have definitely voted to do so beginning this fall.

(*c*) Eighteen state licensing boards have adopted the requirement of one or two years' of college work as their minimum standard of preliminary education, and three other states will have adopted that requirement before the summer is over. Of these twenty-one states, seven require two years of collegiate work as the minimum standard.

(*d*) The licensing boards of thirty-one states are now using their legal powers to refuse recognition to low-grade medical colleges, leaving eighteen boards which do not have legal authority so to do, or which are not utilizing that power.

(*e*) In four more states, New York, Texas, Pennsylvania and Georgia, single boards of medical examiners have been secured replacing the old arrangement whereby the authority to enforce the requirements of the practice act had been parcelled out to three separate and often conflicting boards. Single boards are still seriously needed in three or four states chiefly in Arkansas and Florida.

(*f*) Great improvements have been made in the administration of entrance requirements of medical colleges, nevertheless, it is now clearly known that the chief remaining evil is in connection with the so-called "equivalent" examination. Entrance qualifications in this respect need to be better safeguarded.

(*g*) Three general classifications have been reported by the Council in 1907, 1910 and 1913, respectively. The last has been revised in regard to the ratings of several colleges during the last year.

(*h*) The present classification of colleges is based on a ten years' study of medical education including four inspections of practically all medical colleges. The ratings, therefore, are not a matter of guess work, but are based on reliable data which are on file.

(*i*) Twenty-eight states are now supporting medical education and in fifteen of these states the only medical school in the state is the medical department of the state university. There are only ten states having medical colleges in which the states do not contribute directly to the support of medical education.

Other portions of the report are summarized as follows:

(*j*) There needs to be a reorganization of the clinical departments in our medical schools, whereby clinical teaching may be placed on a more satisfactory basis. Toward this end a committee composed of ten prominent clinicians has been appointed who will bring in a report on this important subject at the next conference of the Council.

(*k*) Investigations shows that at the present time there are enough good hospitals in this country to provide internships for all graduates of medical schools, and the sentiment of all the better medical schools favors the adoption by state boards of a requirement that before he is eligible for a license every candidate must have served such an internship. It is suggested that such a requirement if adopted by state boards should be made to apply to graduates of 1918 or 1919 and thereafter.

(*l*) A careful study of graduate medical instruction has been started by a special committee of the Council. The problem differs greatly from that of undergraduate instruction. Admission requirements should be that "any earnest, reputable, licensed practitioner of medicine," would be allowed to take such courses as he is qualified for by previous training and experience. There should be definite regulations in regard to the granting of certificates so as to prevent any suggestion of these schools being diploma mills. These schools eventually like undergraduate medical schools should be developed as departments of universities.

(*m*) The development of graduate medical education is one of the immediate and pressing needs. There is an abundance of clinical material in our large cities which should be developed so that graduate courses may be taken by physicians who cannot spare the time and money necessary to go abroad. The development of graduate medical education is the next great step in the provision for a better trained medical profession in this country.—*Journal A. M. A.*, July 4, 1914.

*Teeth and the Dangerous Trades.* The part played by carious teeth in occupational diseases is no small one. In cases of lead poisoning the point of entry for the destructive agents is often a single carious tooth.

Workers in pits and mines where mercury ores are found, or in mercury distilleries, are perennially exposed to the danger of contracting severe and painful inflammations of the mucous membrane of the mouth, with the inevitably following difficulties in the assimilation of food. Only those with perfectly sound teeth should be allowed to enter trades where mercury is dealt with, and their teeth should be regularly examined so that any tendency to decay may be checked at the outset.

Copper is less dangerous than the above-mentioned metals but will sometimes cause stomatitis. To clorine the buccal cavity is particularly sensitive, and the workers in soda factories are especially prone to dental troubles owing to the action of muriatic acid. The minute particles of meal filling the air breathed by bakers and pastry cooks is most dangerous to the teeth. The percentage of decayed teeth amongst men, under forty years of age, employed in bakeries, is seventy-eight. The gastric and pulmonary diseases so commonly associated with these callings are undoubtedly favored in their inception by the presence of carious teeth and unhealthy gums.

Sulphuric, muriatic and nitric acids attack the front teeth to begin with; mercury and phosphorus the bones, gums and nerves.

Hat-making carries a wide margin of liability to mercurial poisoning.

The initial symptoms of profuse salivation and inflammation of the oral cavity lead to the trembling of the extremities and finally to paralysis. Leppe states that twenty out of thirty hat makers have diseased gums and teeth. Of eighty-one workmen examined, seventeen presented symptoms of mercury poisoning. Painters show a high percentage of carious teeth. Twenty-two members of the Strassburg Benefit Society, painters, made up between them, 1902, 1904, one hundred and sixty-five days of absence from work wholly on account of dental trouble.

The typical professional malady of glass blowers is a syphilitic affection of the mouth provoked by the common use of several workmen of one blow pipe. The peculiarity of the disease in these cases is that it flourishes for a long time unseen in the depths of the oral cavity, and a number of persons may be infected before its presence is discovered.

The prophylaxis and therapeutics of dentistry for the dangerous trades should include advice as to the choice of specialties for the mouth. Generally speaking pastes are to be preferred to powders for those whose mouths are daily in danger. Preparations packed in lead tubes should be rigorously excluded. Soft, not stiff, tooth brushes should be used. Mouth washes should be astringent rather than acid. —*Abstracted from the Deutsche Zahnarztliche Zeitung.*—By Dr. Hanauer, in *Oral Health.*

*A Good Syringe.* Take an ear syringe. It is a rubber bulb with a long integral point. They can be obtained at a drug store for 25 cents. Take a glass tube that fits the point of bulb on the outside. Hold the glass in the Bunsen, and draw it in the center, so as to make two pieces each with a sharp point. Cut with a file, and snap off.

You have two pieces now, and take one and hold in the burner until it droops, and the hole is small. Put it on the tube of syringe bulb, and you will find that it will not leak if rightly adjusted.

This will require some little experiment, but when made, a good syringe is had that will hold peroxide, carbolic acid, or anything that would injure metal or rubber. It can be used in any way, and sterilized in an alcohol lamp, by dipping the point in the alcohol, and allowing it to burn off. It will sputter and burn in the tube, but in no case have I found it break the tube.

There is possibly no way known more effective to destroy germs than the above.—*Northwest Journal.*

*Casting Aluminum.* Aluminum is a most peculiar and refractory metal, with warpage or shrinkage, which is, of course, due to the readjustment or rearrangement of the molecules during and after crystallization, is probably to be diminished or overcome only by alloying or by the use of a very high grade of investment material.

With any of the investment materials now used for this purpose, it does not seem to matter how rapidly or how slowly the case is cooled after the casting is made; whether it is plunged into cold water immediately after crystallizing, or allowed to stand until thoroughly cold, or even for a day or so afterward, it will be noted that as soon as the case is removed from the investment and the resistance offered thereby is released, the molecules will at once rearrange and readjust themselves, which is to be observed by holding the casting close to the ear and noting the musical sounds which are easily discernible.—Hart J. Goslee, *in Dental Review.*

*Indications for the Amount of Oxygen Required in Nitrous Oxid and Oxygen Anesthesia.* Since the amount of oxygen in the mixture of nitrous oxid and oxygen controls the depth and character of anesthesia, we are constantly required to determine whether too much or too little oxygen is being used. The following symptoms—no one of which is infallible, of course—aid in this determination.

Too little oxygen may produce—Muscular twitching, becoming clonic spasms or jactitation; panting respirations; retching or vomiting; progressive cyanosis; sudden blanching (rarely); dilated pupils; spastic rigidity of muscles; hesitating respiration with prolonged expiration; cessation of respiration from muscular spasm.

Too much oxygen may produce—Voluntary or reflex resistance; superficial slow respiration; retching or vomiting (rarely); increasing pink, becoming bright; active pupillary reflex; reflex resistance about incision; prolonged inspiration; holding of breath; talking, crying, etc.

Pulse and respiration are usually accelerated when the patient is under the influence of nitrous oxid and oxygen, but when these gases are properly administered the blood pressure is not influenced. The volume of respiration or the amount of gas inhaled at each breath is subject to considerable variation, but in the average case amounts to 500 cc. About thirty respirations per minute is the most frequent rate. Neither the rate nor the volume of respiration means much in itself, but the amount of gas consumed per minute, which represents the ventilation of the lungs, is of importance.—E. I. McKesson, *Summary and Cosmos.*

*Ten Hygienic Truths.* The late Dr. Frank H. Hamilton, of Bellevue Hospital, is said to have framed the following curious decalogue of health precepts:

"1. The best thing for the inside of a man is the outside of a horse.

"2. Blessed is he who invented sleep; but thrice blessed the man who will invent a cure for thinking.

"3. Light gives a bronzed or tan color to the skin; but where it uproots the lily it plants the rose.

"4. The lives of most men are in their own hands, and, as a general rule, the just verdict after death would be, *felo de se.*

"5. Health must be earned—it can seldom be bought.

"6. A change of air is less valuable than a change of scene. The air is changed every time the wind is changed.

"7. Mould and decaying vegetables in a cellar weave shrouds for the upper chambers.

"8. Dirt, debauchery, disease and death are successive links in the same chain.

"9. Calesthenics may be very genteel and romping very ungenteel, but one is the shadow, the other the substance, of healthful exercise.

"10. Girls need health as much—nay, more than boys. They can only obtain it as boys do, by running, tumbling—by all sorts of innocent vagrancy. At least once a day girls should have their halters taken off, the bars let down and be turned loose like young colts."—*Health.*

*Who Can Take an Anesthetic*

Modern surgery has robbed the most difficult surgical procedure of its dangers. The surgeon wizard of to-day can enter any of the body cavities with comparative safety and perform feats of dexterity little short of marvelous. Yet any operation, however slight, is fraught with danger to life, owing to two incidental factors—surgical shock and anesthesia. Even in the hands of experts an anesthetic may prove fatal in a given case, the possibility of such a calamity being in inverse proportion to the experience of the anesthetist. No wonder the patient dreads the anesthetic, while the surgeon is continuously hunting for a safe agent which is yet to be found. While it is generally true that ether is safer than chloroform, yet some surgeons have operated on hundreds of patients under chloroform without a single fatality, while others have experienced fatal results from ether. Generally, the contra-indications to general anesthesia are known, yet in a given case it is difficult to foretell the possible effect of the anesthetic. Patients with valvular heart disease, renal disease, atheroma, and other conditions which are supposed to contra-indicate the use of a general anesthetic, have been carried through even prolonged anesthesia safely. One thing however, is certain, viz, that general anesthesia is particularly dangerous to persons suffering from myocardiac changes, a condition which does not permit of ready recognition. For this reason the simple test suggested by Dr. W. A. Schtange in *Roussky Vratch*, January 18, 1914, will be welcomed by the anesthetist, if further experience should verify the doctor's assertions. This test is based on the fact that a healthy person can suspend breathing from thirty to forty seconds. This the author states, depends, not on the lung capacity, but on the vigor of the heart muscle. In persons with weak hearts, the time is shortened to twenty or even ten seconds. The patient, seated in a chair, is told to make a moderately deep inspiration and with the mouth closed to hold his breath as long as he can. The shorter the time the patient can suspend breathing, the greater the danger of an anesthetic, the latter being contra-indicated if the time is less than twenty seconds.

[This Schtange test should prove of great value to the dentist, before inducing general anesthesia by nitrous oxid and oxygen anesthesia, or local anesthesia, especially because unfortunately, most patients are loath to undergo a thorough physical examination by the dentist.]—*Cosmos*, Sept. 1914, page 1091.

*A Broader and Better Dental College.* Fewer and better institutions are what the Profession wants today. An institution, so high in its ideals, so conscientious in its work, so jealous of the interests and welfare of the student that he who graduates from that ininstitution will be a man of high morals, high ideals, well grounded in theory and highly proficient in technique; not only a dentist, but a man of the highest order possible.

The technique as required today in the better Dental Colleges is in most respects quite sufficient, provided the student has diligently endeavored to avail himself of all his opportunities to perfect and broaden his clinical experience. On the other hand, in spite of the cry of the student that he is having too much unnecessary theory, he is getting too little, and should be given a more thorough course in most of the subjects, included in the curriculum, such as Materia Medica, Therapeutics, Histology, Pathology, Physiology, Anesthesia, Medical Diagnosis, and last but not least, a thorough business course.

What a satisfaction and unlimited help it would be to the dentist if he were familiar with the diseases of the soft tissues of the oral cavity, if he could diagnose, differentiate and prescribe for the different *lesions* of the soft tissues, or the closely related *systemic* diseases.

All these things and more are coming, and nothing short of a four-year's course of instruction is sufficient.

Let me also mention the moral and ethical atmosphere and the great part each plays in the development of the student. If the student body is to respect its faculty the respect must be prompted by something more than excellence in technique or theory of the individual members of that faculty. There must be some moral standard, some high ideal held by each member or by the faculty as a whole that will command, rather than demand, the respect of the "student body" and instill in each one the desire to develop these morals and ideals.—Dr. G. M. Crow, in *Northwest Journal of Dentistry.*

*Syndicalized Surgery Prophesied.* That an essential change in the relation of doctors to the public is to be expected in the near future has been asserted by many who have given thoughtful attention to the steady advance in recognized importance of preventive, as compared with curative medicine. This creates what on high authority has been declared to be an irresistible tendency toward making the physician a salaried official, serving communities rather than individuals.

Preparatory for that alteration of status, or rather for its completion, for it is already well begun, there will come, according to *The American Journal of Surgery*, an economic change in the relation of doctors to each other. The day is far past, it says, since the family physician supplied all there was of both medical and surgical skill. Now the original divisions of practice have been subdivided, for specialism has developed in both branches of the profession. But while the line between medical and surgical diseases is sharply defined, that between medical and surgical patients is fading away, and in few instances can the surgeon, any more than the doctor, work alone. He must often call in other specialists, such as the radiographist, the pastro-enterologist, the urologist, and the bacteriologist, and besides these there are the nurse, the historian, and the anesthetist, whose help may be necessary.

But, declares The Journal, while "the various scientifically co-ordinated specialties remain economically separate business enterprises, defeat of their best possibilities is invited." This means, it adds in conclusion, that either the members of the medical profession must go into partnership with one another or they must go into partnership with the public. Either economic competition must fall in line and give place to economic co-operation, just as we have scientific co-operation, or the public must confiscate the medical profession, as it is now proceeding to do in England. The surgeon must syndicalize himself or the public is going to socialize him. One or the other of these is inevitable. Either is to be preferred to the present competitive system, in which both surgeon and patient are the victims of economic maladjustment.—*The Dental Outlook.*

*Polishing Aluminum.* Aluminum may be scraped the same as vulcanite, but it may be dressed down easier with the emery cloth bands on the lathe arbor. The lathe should be run at high speed, and light pressure used in holding the work against the abradant. Scratches are removed from the surface by the use of felt buffers and pumice, followed with a soft brush and whiting. This gives the aluminum a leaden polish due to fine particles of the metal and abradant being ground into the surface of the metal base-plate. There are three methods of cleansing and developing the luster of aluminum—(1) chemical, (2) use of special polishing preparation, and (3) burnishing. The chemical method consists of treating with caustic alkali and acids alternately. This method is hardly suitable for the dental laboratory. However, it gives a beautiful frosted effect, but it is doubtful if it is desirable.

There are several preparations useful for the second method. Benzine and whiting rubbed on with a buffer of cotton or chamois skin, or these materials used by hand. A paste may be made of two drams each of copper sulphate and potassium carbonate, and one ounce of lard. To this paste add a dram or two of any one of several finely ground abradants, as chalk, rotten stone, rouge, tripoli, or buckhorn.

The burnishing is done with an agate or bloodstone burnisher and a burnishing fluid composed of equal parts of olive oil and rum. This method is the very best for the maxillary and mandibular surfaces, but is open to the objection that is shows up the unequal hardness of the dressed surfaces. However, it produces the hardest and best wearing surface of any of the methods for finishing.—Geo. H. Wilson, in Oct. *Summary*.

*Speed in Surgery under Anesthetics.* One often hears the statement, and in talking the matter over with the patients they often express the opinion, that when they are in that state the operator can work much faster than he could were they to feel the pain. This should never be done. Just as much care should be taken by the operator with reference to the use of sharp instruments as if the patient felt the pain; nor should he use the burr any longer, nor push the work any harder, than if he was working on a patient in the normal state who is not over-sensitive. The shock to the pulp, while not felt, is just as great, and the danger exists that too rapid work will be followed by pulpitis, and even death of the pulp and later abscess. I was impressed, even before using this method, with the facility with which a pulp could be damaged, and still I remember that in my early days I had a case of pulpitis develop after a cavity preparation under anelgesia, in spite of what I then considered ample precautions against overheating. And so I use this method, not so much with a view of saving time, except, perhaps, in the nervous and hypersensitive, but rather for the purpose of eliminating fear and anticipation and avoiding pain and shock. One administration usually suffices to make the patient feel at home with it, and after that they demand it. Men usually express themselves as being ready for the next jag. Women enjoy the perfect rest of mind and body which comes with it, and both glory in the absolute loss of fear and anticipation of pain, as well as the pain itself, which marked their previous sittings in the dental chair.—Dr. Schultz, in *Review*.

*Neothesin—A new Obtunder of Sensitive Dentine.* I have known for some time that formaldehyde would desensitize dentin. The problem that I have been trying to solve has not been to obtund sensitive dentin. This can be accomplished in many ways, but to obtund hypersensitive dentin to a given depth so that the pulp of the tooth will not be affected has been the problem. Formalde-hyde is an irritating agent; it is hard to handle. Some are so afraid of it that they would not even seal it in the canal of a putrescent tooth. How to harness up this gas and make it do its work has been somewhat of a problem. We had to modify the irritating effect of formaldehyde. Novocain was too slow in its action. Those who use novocain have admitted tonight that they have to wait eight or ten minutes after injection in order to have the anesthesia produced. I had to have a remedy that would act upon the sensitive fibrils as soon as it was applied. Cocain will do that much more readily than novocain, and yet cocain, rapid as it is, is a little slow. A new local anesthetic has been developed recently. You have not heard anything about it, to any appreciable extent at least, becasue experiments now are being conducted with it. It is an anesthetic called neothesin, and is supposed, according to the experiments thus far, to be equal in anesthetizing power and twice as rapid as cocain. I can take neothesin, combine it with trioxmethylen, which is solid formaldehyde, add to it a little thymol, for its disinfectant part, and I have a remedy that is absolutely certain and absolutely safe. The proportion of these three ingredients is 11 parts of neothesin, 12 of thymol, and 77 of trioxmethylen. I take those three drugs and combine them with a petrolatum base, coloring with some insoluble material to tell it from the tissue structures, and one grain of that combination is enough for about fifteen to twenty applications. Reasoning on the basis of fifteen applications to one grain of the combination, we have in that one application 1-300 of a grain of neothesin, 1-270 grain of thymol, and 1-43 grain of trioxmethylen. You can take the most sensitive cavities that you have dreaded to see come in—gingival cavities—seal that remedy in for twenty-four or forty-eight hours, and you can absolutely drill to your heart's content, providing, of course, you don't drill too far. You can't go to the pulp and expose it, and I am glad of it. This remedy, fortunately, is automatic and practically fool-proof, in that if you place it in the tooth where contra-indicated it will make it ache. That is one of the best things about it, because that is a check on the careless operator who would use it in those cases where the pulp, because of its pathology, should be removed.—Dr. Buckley, in *Review*.

*Contact Points.* I wish to emphasize the fact that the contact point is frequently placed very colse to the occlusal, instead of leaving it a little down from the occlusal where it is normally. The occlusal surfaces of both teeth should be inclined a little so as to invite food to glide into the interproximal space, then if the contact is right part of it will pass out to the buccal and part of it will pass out to the lingual and in that way will scour the proximal surfaces. Just in proportion as you raise the contact and thereby flatten the occlusal surface away from that contact, you tend to have food diverted from the interproximal space and thus lose the cleaning effect. The area of liability to decay on such surfaces is thereby increased. I don't know why these contacts have been put higher, but that seems to be the general tendency.—A. D. Black, in *Western Journal.*

